# Perihematomal brain tissue iron concentration measurement by MRI in patients with intracerebral hemorrhage

**DOI:** 10.1111/cns.13395

**Published:** 2020-05-21

**Authors:** Jialiang Wei, Nemanja Novakovic, Thomas L. Chenevert, Guohua Xi, Richard F. Keep, Aditya S. Pandey, Neeraj Chaudhary

**Affiliations:** ^1^ Department of Neurosurgery 1500 E Medical Center Dr Ann Arbor MI USA; ^2^ Department of Radiology 1500 E Medical Center Dr Ann Arbor MI USA

**Keywords:** brain edema, intracerebral hemorrhage, iron, magnetic resonance imaging, relaxivity maps

## Abstract

**Aims:**

Over the past two decades, animal intracerebral hemorrhage (ICH) model studies have indicated that iron, released after hemoglobin degradation, is neurotoxic. Iron phantom and animal experiments have shown that magnetic resonance imaging (MRI) relaxivity maps correlate with iron concentration. This study expands this into patients.

**Methods:**

Eighteen human subjects with ICH underwent MRI at 3, 14, and 30 days. R2* relaxivity maps were used to calculate perihematomal iron concentrations and T2 imaging to determine hematoma and edema volumes.

**Results:**

Perihematomal iron concentrations were increased at all three time points and decreased with distance from the hematoma. While perihematomal iron concentrations did not vary with hematoma size, the total iron overload (increased iron concentration x volume of affected tissue) did. Total iron overload correlated with edema volume.

**Conclusions:**

These results demonstrate the feasibility of measuring perihematomal iron in ICH patients which may be important for monitoring treatment strategies and assessing efficacy noninvasively.

## 1 INTRODUCTION

Intracerebral hemorrhage (ICH) has devastating consequences.^1^ Neural damage following ICH is due to mechanical disruption plus secondary injury due to red blood cell lysis and hemoglobin degradation products.^2^, ^3^ Animal ICH models have implicated iron as a major mediator of neurotoxicity with protection with an iron chelator, deferoxamine.^2^, ^4^

There are no FDA approved therapies for ICH.^5^ Recently, MISTIE III, an image guided minimally invasive hematoma evacuation randomized to conservative management trial, demonstrated a trend to improved outcome in the evacuated group.^6^ In addition, iDEF, a phase II trial assessing optimal dose of deferoxamine in ICH needed for improved outcome, demonstrated a trend to improved outcome at 6 months from ictus.^7^ However, both the above trials failed to demonstrate statistically significant benefit with respect to their primary endpoints. Probably, there are other iron related factors at play at the cellular level which directs extent of neuronal injury and are not optimally mitigated by deferoxamine therapy, as has been demonstrated in some other rat ICH model studies.^8^

Once relaxivity maps (R2* which equals 1/T2*) are created on MRI, regions of interest measurement can be performed enabling calculation of iron concentration based on corresponding susceptibility in iron phantoms.^9^ The proof of principle and concept validation was also examined in an animal model.^10^ The current study extends this to assessing perihematomal iron concentrations in 18 ICH patients. Neither treatment nor a robust objective noninvasive surrogate marker currently exists. Our aim is to validate one such MRI based iron quantification algorithm in the human population.

## 2 METHODS

### 2.1 Patients and MRI

This study was approved by the Institutional Review Board of the University of Michigan. Eighteen patients were recruited to the study based on inclusion and exclusion criteria following screening of all ICH patients and written informed consents. To be eligible, patients had to be 18 to 85 years old with a spontaneous basal ganglia hemorrhage, where it was safe to obtain a noncontrast MRI brain at days 3, 14, and 30. The exclusion criteria included hemorrhage caused by intracranial aneurysm, arteriovenous malformation, trauma, hemorrhage into ischemic stroke, brain tumor, brain calcification, thrombocytopenia, or coagulopathy of any kind, patients in whom treatment and/or life support is already being withdrawn at the time of enrollment, patients with decreased hepatic function (defined as aspartate aminotransferase or alanine aminotransferase >2.5 times the upper limit of normal), bilateral hemorrhage, patients in whom surgical evacuation of hematoma is planned at the time of enrollment, patient age <18 years, or pregnancy. The 3 Tesla MRI sequence used for iron quantification was 3D TR = 40 ms, TE = 6.5, 11, 15.5, 20, 24.5, 29, 33.5, 38 ms, 1.5 mm slice‐to‐slice, acquired as 3 mm, acquired resolution matrix = 240 × 240, FOV – 240 mm × 240 mm. Standard T1‐ and T2‐weighted sequences were used to assess hematoma and edema size. R2* maps were then created using Matlab.

### 2.2 Image analysis

Images were analyzed using 3D slicer software (slicer.org). MRI physicist helped to create the matlab files for R2* images which then were used to calculate iron concentration. In addition, images of T2 and FLAIR matlab files were also utilized for calculation. All the slices which demonstrated signal abnormality and the hematoma or the abnormal T2 or FLAIR signal were included in the calculation. Briefly, three concentric rings were drawn surrounding the hematoma at days 3, 14, and 30. The 1st ring was conducted on the outer part of the high‐signal ring around the hematoma, and the 2nd and 3rd rings were drawn outwardly from the previous rings. The width of each of ring was 2 mm. Control iron calculations were conducted using contralateral cortex, basal ganglia, and white matter. Total iron overload (IO; mg) was calculated by comparing ipsi‐ and contralateral iron concentrations multiplied by the volume of the rings: IO = ((ring 1 [Fe]‐contr [Fe]*(ring 1 volume)) + ((ring 2 [Fe]‐contr [Fe]*(ring 2 volume)) + ((ring 3 [Fe]‐contr [Fe]*(ring 3 volume)).

### 2.3 Statistics

Data which were normally distributed (day 3) were analyzed by ANOVA with Tukey's post hoc test and are presented as mean ± SD. Non‐normally distributed data (days 14 and 30) were analyzed by Kruskal‐Wallis test with a Dunn's post hoc and are presented as medians. The relation between iron overload (days 3, 14, and 30) and hematoma volume or brain edema (days 3 and 14) was analyzed by regression analysis. Differences were considered significant at *P* < .05. The analysis was performed with and without the one outlying data points in Figures 2 and 3, and the correlation was upheld.

## 3 RESULTS

**FIGURE 1:**
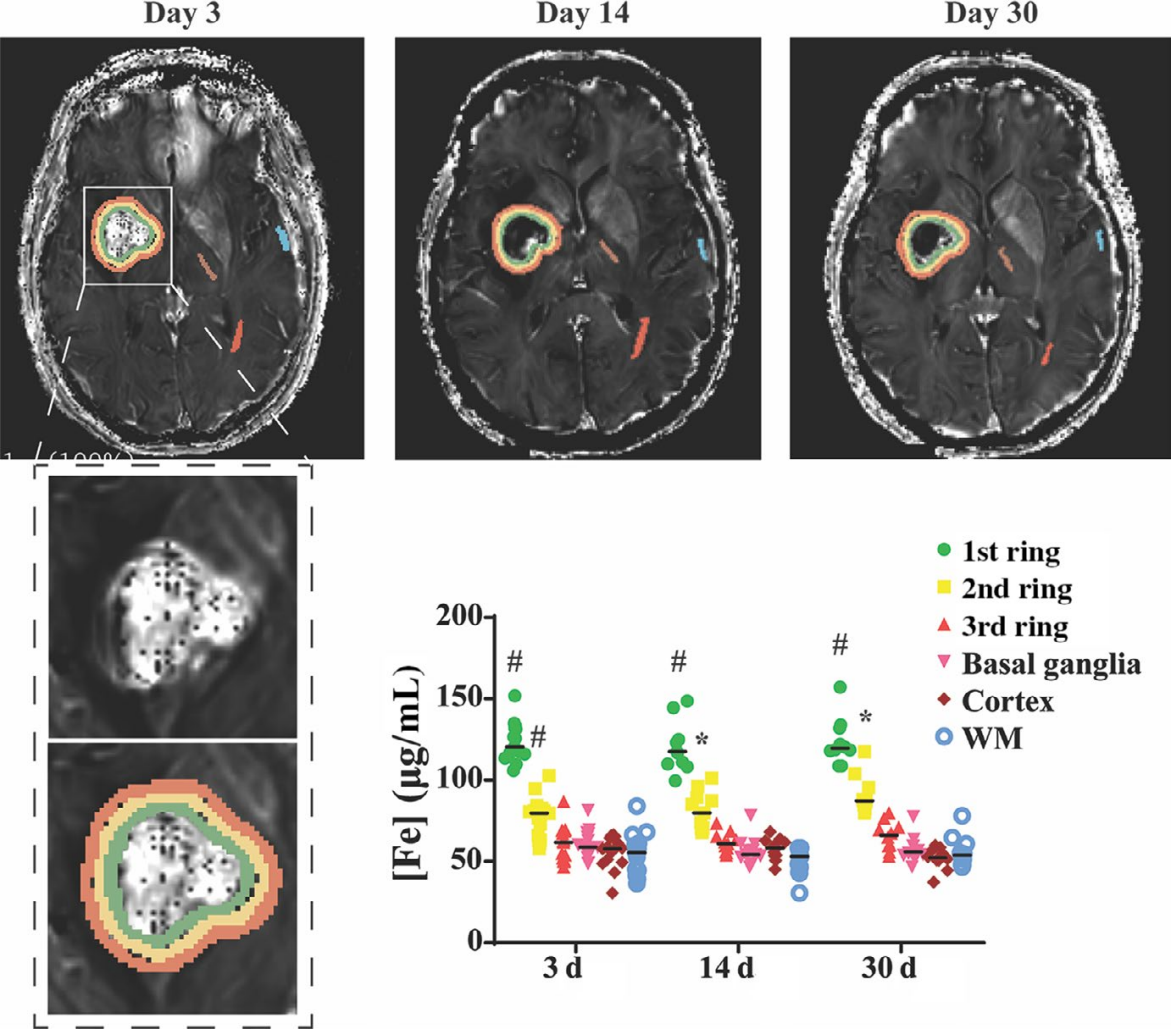
Representative examples of MRIs used to determine perihematomal iron concentrations at 3, 14, and 30 d after ICH. Three concentric rings (green, yellow, and orange) were drawn around the hematoma and iron concentrations determined in each ring as well as contralateral basal ganglia, cortex, and white matter. The graph shows the median perihematomal iron concentration in each ring with time along with contralateral concentrations. Values are median, n = 18, *P < .05 vs contralateral basal ganglia, #*P* < .001 vs contralateral basal ganglia

Eighteen recruited patients (Male:Female = 8:10) were 22‐84 years old. Eight patients had all 3 data points collected at days 3, 14, and 30. Six patients had only day 3 MRI. Two of them had day 14 and day 30, and another two had day 3 and day 14. The lack of a complete dataset in all patients is a combination of loss to follow‐up and patient family refusal to continue with participation in the study. Hematoma volume at day 3 was 0.64‐34 mL (Table. 1). Perihematomal iron concentrations, as assessed on R2* images, increased after ICH. At days 3, 14, and 30, the iron concentration in ring 1 (eg, Day 3:124 ± 11 vs 60 ± 8 µg/mL; *P* < .001 vs contralateral) and ring 2 (eg, Day 3:77 ± 12 µg/mL; *P* < .001 vs contralateral) was significantly elevated, though the iron concentration in ring 2 was less elevated than in ring 1. The ring 3 iron concentration was not significantly different from contralateral at days 3, 14, and 30 (Figure 1). The F value on the day 3 comparisons was F (3, 42) = 181.8. Median value differences were 61.7 (1st ring vs basal ganglia at day 3), 21.09 (2nd ring vs basal ganglia at day 3), 3.08 (3rd ring vs basal ganglia at day 3), 63.29 (1st ring vs basal ganglia at day 14), 25.64 (2nd ring vs basal ganglia at day 14), 6.68 (3rd ring vs basal ganglia at day 14), 64.86 (1st ring vs basal ganglia at day 30), 32.65 (2nd ring vs basal ganglia at day 30), and 11.48 (3rd ring vs basal ganglia at day 30).

**TABLE 1:** Demographics and time points of MRI scans with hematoma volume

Age	Gender	Day 3	Hematoma volume (cc)	Day 14	Hematoma volume (cc)	Day 30	Hematoma volume (cc)
69	F	+	5.7	−	−	−	−
54	M	+	33.9	−	−	−	−
57	M	+	13.4	−	−	−	−
69	F	−	−	+	22.2	+	27.1
51	M	−	−	+	2.1	+	1.8
48	F	+	4.9	+	5.1	+	4.7
56	M	+	0.6	+	0.2	+	0.15
69	M	+	5.1	+	7.2	+	6.9
25	M	+	34.3	+	28.6	−	−
63	F	+	9.3	+	7.4	−	−
22	F	+	24.3	+	21.7	+	19.4
38	F	+	4.1	+	4.0	+	3.9
73	F	+	1.9	−	−	−	−
84	F	+	5.1	+	5.0	+	3.2
65	M	+	15.7	−	−	−	−
65	F	+	14.6	+	19.2	+	8.8
58	F	+	17.5	−	−	−	−
67	M	+	24.8	+	26.4	+	16.9

**FIGURE 2:**
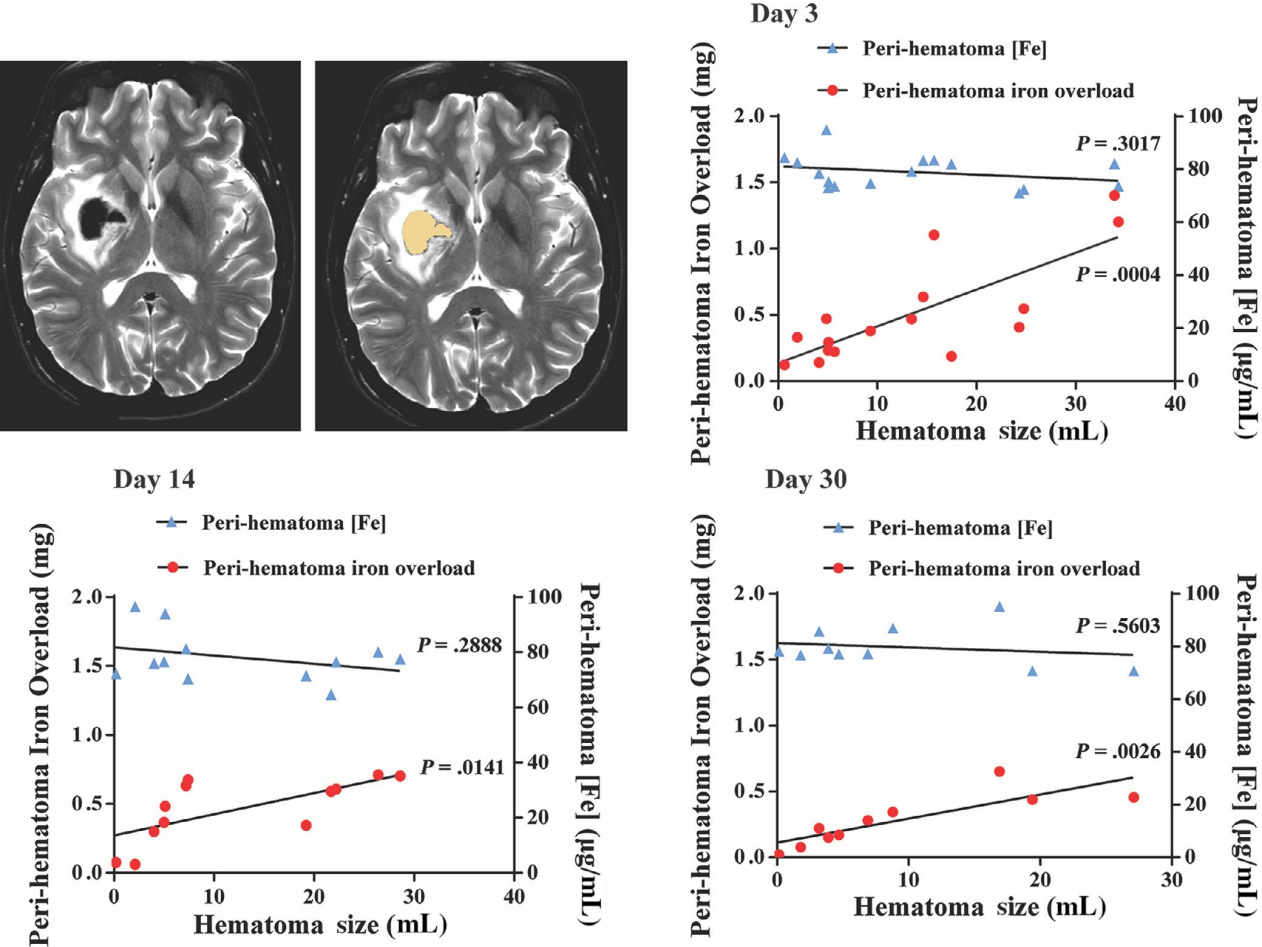
Representative T2‐weighted MRI used to calculate hematoma volume. The hematoma is delineated in yellow (2nd image). The graph shows the relationship between hematoma volume and both perihematomal iron overload and iron concentration at days 3, 14, and 30. There was a significant correlation between hematoma volume and overall iron overload at all three time points (day 3: *r* ^2^ = .6014, *P* = .0004; day 14: *r* ^2^ = .4682, *P* = .0141; day 30: *r* ^2^ = .6980, *P* = .0026) but not with perihematomal iron concentration at any time points (day 3: *r* ^2^ = .0759, *P* = .3017; day 14: *r* ^2^ = .1115, *P* = .2888; day 30: Spearman *R*: −.2121, *P* = .5603)

**FIGURE 3:**
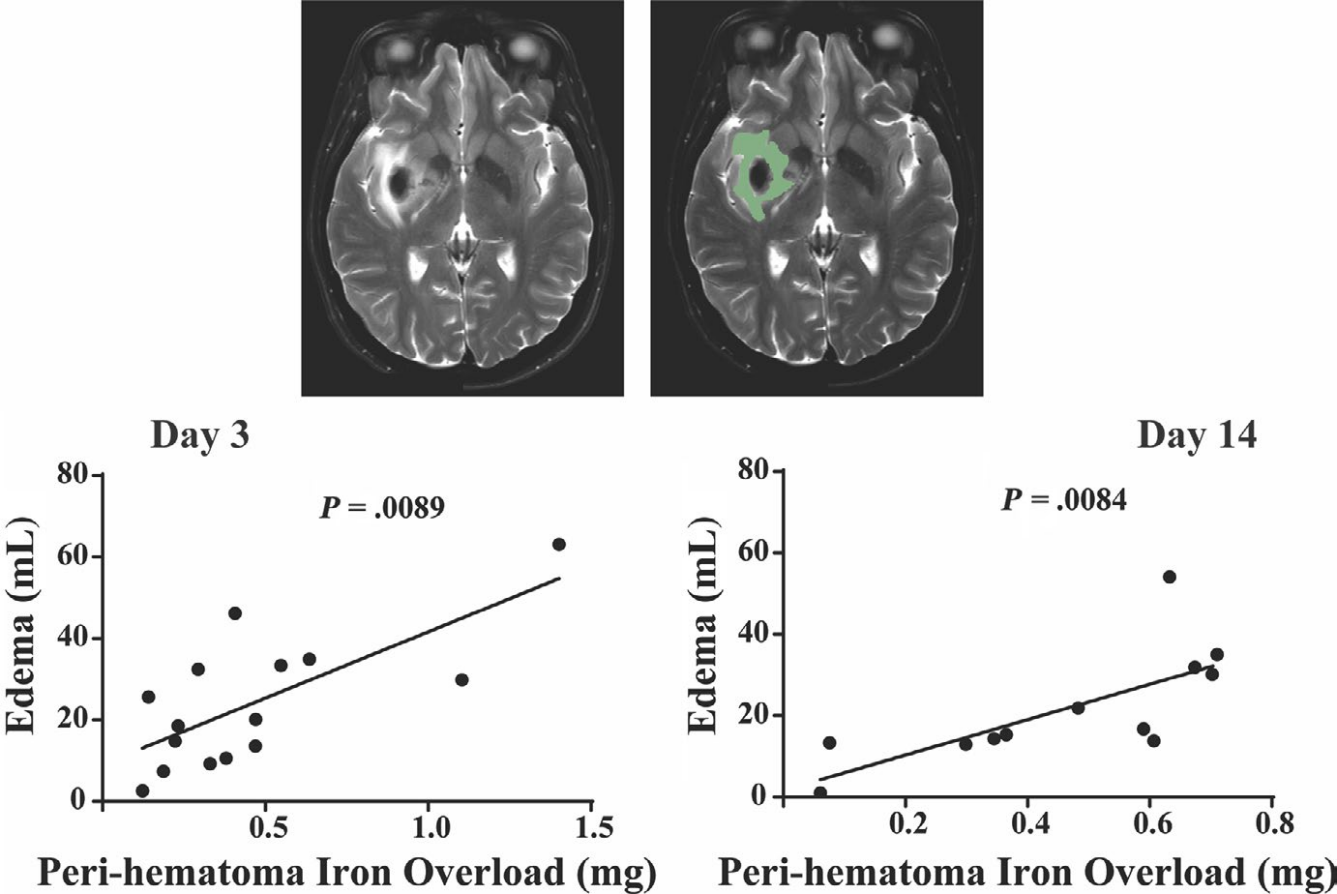
Representative MRI T2‐weighted image showing perihematomal edema which is delineated in green in the second image. The graph plots edema volume against perihematomal iron overload (IO) in individual patients at days 3 and 14. There was a close correlation at days 3 (Spearman *R*: .6607; *P* = .0089) and 14 (*r* ^2^ = .5174; *P* = .0084)

Iron overload (IO) in the perihematomal tissue was calculated based on difference in iron concentration between ipsilateral and contralateral tissue and the volume of the measured rings (see Methods). There was a positive correlation between IO and hematoma size at days 3, 14, and 30 after ICH (day 3: *r* ^2^ = .6014, *P* = .0004; day 14: *r* ^2^ = .4682, *P* = .0141; day 30: *r* ^2^ = .6980, *P* = .0026; Figure 2). In contrast, the iron concentration in three rings was relatively constant across different hematoma sizes (day 3: *r* ^2^ = .0759, *P* = .3017; day 14: *r* ^2^ = .1115, *P* = .2888; day 30: Spearman *R*: −.2121, *P* = .5603; Figure 2). At day 3 and day 14, edema volumes on T2‐weighted images correlated with IO (day 3: Spearman *R*: .6607, *P* = .0089; day 14: *r* ^2^ = .5174, *P* = .0084; Figure 3).

## 4 DISCUSSION

This study demonstrates that MRI with R2* mapping is a robust way to track tissue iron levels in the immediate periphery of the hematoma over a period of 30 days following hemorrhage. It demonstrates a correlation between the IO in the perihematomal tissue with hematoma size but interestingly, no correlation of perihematomal iron concentration at set distances from the hematoma and hematoma size. Perihematomal edema volume correlated with IO.

The concept of measuring iron concentration by MRI signal magnitude calculations has been validated by biochemical assay in a rat ICH model.^4^ The human concentrations correlate well with those obtained in rat and porcine ICH models.^10^ Other animal studies have also quantified iron with Raman spectroscopy and X‐ray fluorescence demonstrating iron concentration in the perihematomal regions at days 7, 14, and 21 after ICH.^11^ Their study demonstrated ion dyshomeostasis following initial injury to be the reason for behavioral changes in the animals and that rehab following injury reduced neuronal damage. The iron measurements that were obtained compared well with the levels we have measured in our human ICH study. Contralateral iron concentrations also correlate well with concentrations determined in human brain tissue by other methods.^12^

The current study presents the first 18 ICH patients where perihematomal iron levels were tracked by MRI at multiple time points following ICH with MRI. It demonstrates that perihematomal iron levels were significantly higher than those measured at the same anatomic site in the contralateral hemisphere in ring 1 (0‐2 mm from hematoma edge; iron concentration ~ 120‐124 µg/mL) and ring 2 (2‐4 mm from hematoma edge; iron concentration ~ 77‐80 µg/mL) on days 3 and 14. In ring 3 (4‐6 mm from edge; ~60‐66 µg/mL), compared to contralateral (~57‐60 µg/mL), there is significantly increased levels only on day 30, perhaps indicating a ring of tissue “at risk” from an advancing rim of iron‐induced toxicity. Interestingly, the perihematomal concentrations did not change significantly between 3 and 30 days postictus.

Interestingly, the perihematomal iron concentration did not vary with hematoma size, perhaps suggesting that the increase in iron is related to events at the periphery (rather than deep within) of the hematoma. In contrast, the total perihematomal iron overload (µg/Ml × volume of affected tissue) was dependent on hematoma size. Total IO at days 3, 14, and 30 correlated well with hematoma size (Figure 2). Our analysis also demonstrates that on days 3 and 14, absolute edema volume correlates well with IO (Figure 3). We did not include day 30 edema level in the assessment as we found that the day 30 MRI T2‐weighted sequence did not demonstrate any significant measurable edema as it had almost completely resolved. Based on the hematoma size and the iron concentration of blood, we estimate that the amount of iron release into the perihematomal area at day 3 is ~5% of the initial hematoma iron content. This assumes that no iron is cleared from the perihematomal region by day 3, an assumption needing verification.

We believe this is the first study using MRI R2* maps in human ICH subjects to demonstrate a tissue level marker that can be reliably tracked with time following ictus. Sun et al^13^ have shown that MRI quantitative susceptibility mapping (QSM) images are a more accurate measure of hematoma size compared to GRE images on MRI. It demonstrates the possibility of MRI to accurately calculate the degree of bulk susceptibility within the hematoma but does not use multiple echo points, as performed in the current study, for a more accurate calculation of the susceptibility magnitude.

Preclinical studies indicate that one element of ICH‐induced brain injury is iron‐mediated.^14^ Two recent clinical trials have employed methods that might reduce such toxicity. The MISTIE III trial used minimally invasive, tPA‐mediated, hematoma removal,^6^ that is, removing the source of iron. The iDEF trial used an iron chelator, deferoxamine, to reduce iron‐mediated toxicity.^7^ Both trials missed their primary endpoints but had some evidence of efficacy. A combination of hematoma evacuation and iron chelate therapy has been proposed as a possibility of combining the benefits of both aspects to improve efficacy.^15^ The current study shows the feasibility of measuring perihematomal iron noninvasively in ICH patients. This methodology may be useful in informing such trials and others in the future as a potential surrogate measure of natural history and efficacy.^16^

There are limitations to our study. (a) It is difficult to be absolutely certain whether we are accurately identifying the hematoma edge. In order to increase accuracy, we utilized anatomical MRI (T1‐ and T2‐weighted) sequences to delineate that edge. This was then superimposed on to the R2* maps. (b) The sample size is relatively small. Due to the nature of human subject enrollment studies, not all patients had all the data points from MRI scans at all the prespecified time points. Future larger studies are needed to confirm correlation of IO with hematoma size and edema volume and to examine longer term changes. In our analysis, there is no way of differentiating ferrous (Fe^2+^) or ferric (Fe^3+^) ion in the lysed and denatured hemoglobin molecule in the intra or extracellular space. We are thinking of developing MR ligands to tag Fe^2+^ and Fe^3+^ ions to study their behavior on MRI in the periphery of the hematoma. There is also no way of identifying which cell types undergo iron overload after ICH by MRI. For example, to which extent is the iron in macrophages, astrocytes, and/or neurons. This is important as iron may have different effects on different cell types.

The ability to measure brain tissue iron by MRI allows an assessment of tissue level events in iron handling after ICH. Future plans include analysis to examine whether the extent of hemolysis that occurs within the hematoma impacts iron loss to the surrounding tissue. In addition, iron levels in white matter tracts adjacent to the hematoma will be correlated with changes in those tracts as assessed based on MRI tractography using diffusion tensor imaging.

## 5 CONCLUSION

Magnetic resonance imaging provides a method to assess tissue iron handling after ICH and its evolution with time. It potentially provides a robust noninvasive objective criterion to monitor future treatment strategies and assess efficacy suggesting future ICH trials should incorporate MRI as a surrogate marker of iron handling based on susceptibility weighted mapping.

## CONFLICT OF INTEREST

The authors declare no conflict of interest.
